# Prevalence of cannabis and medication use by indices of residential urbanicity and deprivation among Ohio cancer patients

**DOI:** 10.1007/s10552-025-01972-x

**Published:** 2025-02-12

**Authors:** Theodore M. Brasky, Shieun Lee, Bella McBride, Alison M. Newton, Ryan D. Baltic, Theodore L. Wagener, Sara Conroy, John L. Hays, Erin E. Stevens, Anita Adib, Jessica L. Krok-Schoen

**Affiliations:** 1 Division of Medical Oncology, The Ohio State University College of Medicine, Columbus, OH 43210 USA; 2 Indiana University School of Nursing, Bloomington, IN 47408 USA; 3 School of Health and Rehabilitation Sciences, The Ohio State University College of Medicine, Columbus, OH 43210 USA; 4 The Ohio State University Comprehensive Cancer Center, Columbus, OH 43210 USA; 5 Center for Perinatal Research, Abigail Wexner Research Institute, Nationwide Children’s Hospital, Columbus, OH 43215 USA; 6 Division of Palliative Medicine, The Ohio State University College of Medicine, Columbus, OH 43210 USA

**Keywords:** Benzodiazepines, Cancer, Cannabis, Deprivation, Disparities, Medication, Urbanicity, Opioids

## Abstract

**Purpose:**

There is increasing interest in the use of cannabis products to alleviate symptom burden among cancer patients. Although data remain limited, some evidence suggests that state legalization of cannabis is associated with reduced opioid use. Indices of area-level social determinants of health may provide insights into the patterns of symptom-managing behaviors in the context of health equity.

**Methods:**

Residential ZIP codes from 854 Ohio residents diagnosed with invasive cancer at an academic cancer center were used to assign rural–urban commuting area (RUCA) codes and social deprivation index (SDI) values. RUCA was categorized as metropolitan and non-metropolitan, and SDI was dichotomized at the median. Participants completed a one-time cannabis-focused questionnaire which included items on medications used to alleviate symptoms.

**Results:**

The prevalence of self-reported cannabis (19% vs. 13%) and opioid use (30% vs. 21%) were higher among patients living in areas of higher social disadvantage vs. lower. No differences were observed for use of benzodiazepines or for any product by residential urbanicity.

**Conclusion:**

Larger, multi-institutional studies with detailed measurement of cannabis and medications and an increased capacity to examine additional social determinants of health are needed to confirm and explain these descriptive findings.

**Supplementary Information:**

The online version contains supplementary material available at 10.1007/s10552-025-01972-x.

## Introduction

Cannabis product use in the USA is evolving, with drastic increases in cannabis use among adults, including cancer patients, observed over the past several decades [1]. Recent studies by the authors and others show approximately 15–19% current cannabis use among cancer patients at academic cancer centers, with sleep, pain management, stress, and appetite/nausea identified as top reasons for its use [2, 3]. Despite the growing prevalence and established correlates of cannabis use (e.g., male, younger age, lower education, higher symptom burden, other adverse health behaviors) identified among cancer patients [4], the degree to which area of residence plays a role in cannabis use in cancer patient populations is not known.

Measures of residential environment, including urbanicity and deprivation, have been used previously to identify disparities in cancer rates, risk factors, and outcomes [5, 6]. In the general population, recent studies suggest that use of cannabis is higher in urban environments and lower in areas of socioeconomic deprivation [7, 8]. However, data on cannabis use by residential environment among cancer patients are lacking. To our knowledge, only one study has examined differences in cannabis use based on urbanicity [9], and none have investigated its use by measures of socioeconomic deprivation in cancer patients. Herein, we aimed to describe the prevalence of cannabis and symptom-palliating medication use, stratified by measures of residential environment among cancer patients of an academic NCI-designated Comprehensive Cancer Center. Potential implications of observed differences in cannabis use by residential environment include the need for palliative care and opportunities for patient and clinician education on cannabis.

## Methods

### Study sample

Between July 2021 and July 2022, 943 adult cancer patients ages 18 or older, with an invasive cancer diagnosis at any anatomic site, who were treated at 8 clinics at the Ohio State University Comprehensive Cancer Center were recruited into an anonymous cross-sectional study. Detailed study methods are reported elsewhere [2]. Briefly, eligible patients were new or returning patients and who were treated or seeking treatment for their cancer in the past 12 months. Patients with a diagnosis of in situ disease were excluded. Eligible patients were identified in the electronic medical record prior to their clinic visit and recruited in clinic or by phone. For the present analysis, we restricted the sample to 854 patients who reported a valid Ohio residential ZIP code. All participants were offered a $10 gift card for their time. Study protocols and procedures were approved by the OSU Institutional Review Board. At the time this study was conducted, Ohio permitted the use of medical marijuana only.

### Data collection

We administered a cannabis-focused questionnaire designed and validated in cancer patients [2, 10] to assess participants’ cannabis, medication use, and tobacco history, as well as sociodemographic and clinical characteristics and area of residence (including county and ZIP code). Participants completed the questionnaire in their exam rooms during their care appointment or online shortly thereafter. Participants were asked whether they currently use cannabis products (including marijuana and cannabidiol products), and whether they were currently using opioid or benzodiazepines. Follow-up questions assessed the frequency, intensity (number of occasions per use day), mode, and duration of cannabis use, as well as utilization of a medical marijuana prescription. Weekly frequency of opioid and benzodiazepine medication use was also collected.

From self-reported residential ZIP code data, we assigned Rural–Urban Commuting Area (RUCA) codes, created by the U.S. Department of Agriculture Economic Research Service [11]. RUCA codes classify U.S. census tracts using measures of population density, urbanization, and daily commuting, to create a composite measure of urbanicity RUCA codes range from 1 (metropolitan areas with primary commuting flow within an urbanized area) to 7 (rural areas with localized primary commuting flow or to tracts outside urban areas or clusters [11]. We classified RUCA codes of 1–3 as metropolitan and RUCA codes ≥ 4 as non-metropolitan. We similarly applied Social Deprivation Index (SDI) scores [12], as a measure of residential socioeconomic deprivation related to health outcomes using participants’ ZIP codes. SDI scores range from 0 to 100, where 0 indicates least deprived and 100 indicates areas of highest deprivation. The SDI is a composite measure of geographic deprivation utilizing seven measures from the American Community Survey that are thought to impact healthcare access [12]. They include % of population below the Federal Poverty Level, % of population aged ≥ 25 years with < 12 years of education, % non-employed among those aged 16–64 years, % of households living in renter-occupied housing units, % of households living in crowded housing units, % single-parent families with dependents aged < 18 years, and % of households with no vehicle [12]. Referencing first SDI scores for all Ohio ZIP codes—prefixes 430 through 459—we dichotomized SDI at the Ohio population median of 37, thereby reflecting more (SDI ≥ 37) and less (SDI < 37) deprived residential environment.

### Data analysis

We examined descriptive summary statistics of all variables to check the amount and possible pattern of missing values and assessed the distribution of each variable against outliers and rare outcomes. Chi-square tests were used to examine differences in cannabis and medication use by measures of geographic urbanicity and socioeconomic deprivation. All analyses were conducted using SAS (version 9.4; Cary, NC).

## Results

Thirty-three and forty-seven percent of participants lived in non-metropolitan and more deprived areas, respectively. Study participants’ sociodemographic and clinical characteristics are presented in **Supplementary Table 1**. The sample consisted of 854 adult Ohio cancer patients with a mean age of 61.5 years (SD = 11.9). Most participants were white (89.2%), attended at least some college (69.8%), and were currently receiving cancer treatment (80.0%). Approximately 56% of the sample was composed of patients recruited from the cancer center’s thoracic (21.1%), breast (17.9%), and gastrointestinal (17.0%) clinics. Eighty percent of patients were currently receiving treatment at the time of recruitment. Generally, participants’ demographic and clinical characteristics were similar by both measures of residential environment. However, there were differences by race, with a larger proportion of Black participants living in metropolitan areas (10.5% vs. 0.7%) and in more socioeconomically deprived environments (11.9% vs. 2.7%).

Overall, the prevalence of current cannabis, opioid, and benzodiazepine use was 16.2%, 25.4%, and 15.5%, respectively. We present their prevalences stratified on categories of RUCA and SDI in Table 1. Use of cannabis, opioids, and benzodiazepines did not differ by level of urbanization of participants’ residences. However, the prevalence of cannabis use (19.1% vs. 13.4%; *p* = 0.03) and opioid use (30% vs. 20.9%; *P* < 0.001) were higher among those living in more versus less socioeconomically deprived areas. When cannabis and opioid use were further examined, individuals living in socioeconomically deprived areas demonstrated higher rates of cannabis use multiple times a day (12.6% vs. 5.9%; *P* < 0.01), use for > 2 years (11.5% vs. 5.9%; *p* = 0.04), and use of medical cannabis without a prescription (14.3% vs. 8.4%; *p* = 0.03) compared to their counterparts. Additionally, cannabis inhalation was more prevalent in areas of greater deprivation. Participants in more deprived areas also reported higher rates of daily opioid use compared to those in less deprived areas (19.8% vs. 11.7%; *P* < 0.01).

**Table 1:** Prevalence of current cannabis and medication use among Ohio cancer patients, stratified on indices of urbanicity and socioeconomic deprivation

	RUCA, n (%)			SDI, n (%)		
Current medication use	Metropolitan (1-3;* n*=568)	Non-metropolitan (≥4;* n*=286)	*P*	Less deprivation (≤37;* n*=445)	More deprivation (>37;* n*=401)	*P*
Cannabis			0.85			0.03
No	471 (83.66)	239 (84.15)		381 (86.59)	323 (80.95)	
Yes	92 (16.34)	45 (15.85)		59 (13.41)	76 (19.05)	
Cannabis type			0.76			<0.01
Non-use	471 (84.11)	239 (84.45)		381 (86.99)	323 (81.36)	
Consumed	32 (5.71)	12 (4.24)		23 (5.25)	20 (5.04)	
Inhaled	26 (4.64)	16 (5.65)		10 (2.28)	32 (8.06)	
Poly-use	31 (5.54)	16 (5.65)		24 (5.48)	22 (5.54)	
Cannabis frequency			0.55			0.08
Non-use	471 (83.66)	239 (84.15)		381 (86.59)	323 (80.95)	
< Daily	54 (9.59)	22 (7.75)		34 (7.73)	41 (10.28)	
Daily	38 (6.75)	23 (8.10)		25 (5.68)	35 (8.77)	
Cannabis intensity, occasions per day			0.62			<0.01
Non-use	471 (83.81)	239 (84.45)		381 (86.79)	323 (81.16)	
1	41 (7.30)	16 (5.65)		32 (7.29)	25 (6.28)	
≥2	50 (8.90)	28 (9.89)		26 (5.92)	50 (12.56)	
Cannabis duration, years			0.49			0.04
Non-use	471 (84.26)	239 (85.05)		381 (86.79)	323 (82.19)	
0-2	41 (7.33)	15 (5.34)		30 (6.83)	25 (6.36)	
>2	47 (8.41)	27 (9.61)		28 (6.38)	45 (11.45)	
Medical marijuana Rx			0.97			0.03
Non-use	471 (83.66)	239 (84.15)		381 (86.59)	323 (80.95)	
Prescription	28 (4.97)	13 (4.58)		37 (8.41)	57 (14.29)	
No prescription	64 (11.37)	32 (11.27)		22 (5.00)	19 (4.76)	
Opioids			0.52			<0.001
No	423 (75.27)	208 (73.24)		349 (79.14)	278 (70.03)	
Yes	139 (24.73)	76 (26.76)		92 (20.86)	119 (29.97)	
Opioid frequency			0.27			<0.01
Non-use	423 (75.00)	208 (72.73)		349 (78.78)	278 (69.67)	
< Daily	61 (10.82)	26 (9.09)		42 (9.48)	42 (10.53)	
Daily	80 (14.18)	52 (18.18)		52 (11.74)	79 (19.80)	
Benzodiazepines			0.60			0.39
No	480 (84.96)	239 (83.57)		379 (85.36)	332 (83.21)	
Yes	85 (15.04)	47 (16.43)		65 (14.64)	67 (16.79)	
Benzodiazepine frequency			0.83			0.59
Non-use	480 (85.26)	239 (83.86)		379 (85.75)	332 (83.42)	
< Daily	40 (7.10)	21 (7.37)		31 (7.01)	30 (7.54)	
Daily	43 (7.64)	25 (8.77)		32 (7.24)	36 (9.05)	

*RUCA* Rural–urban commuting Area, *SDI*, Social deprivation index

## Discussion

This exploratory study sought to describe the prevalence of cannabis and symptom-palliating medication use, stratified by measures of residential environment among cancer patients. Results provide preliminary evidence that use of cannabis and opioids may differ by residential deprivation but not urbanicity. As a measure of urbanicity, RUCA has been utilized in a myriad of studies to determine cancer disparities in incidence, staging, and survival. Individuals living in more rural areas have worse cancer-related outcomes compared to their more metropolitan-dwelling peers [5, 6]. Previous studies have utilized RUCA to explore cannabis use by rurality among the general population [8, 13, 14]. Only Azizoddin et al. [9], examined cannabis use by RUCA status among cancer patients. Like our own findings, the authors reported no differences in cannabis prevalence by RUCA categorized in the same manner as the present analysis [9].

The current study is unique in that it stratified cannabis and medication use by SDI among cancer patients. Previous studies have noted worse outcomes related to cancer incidence, staging, and survival among individuals residing in more deprived environments [15–17]. SDI has been previously utilized as a proxy measure for socioeconomic status and its association with cancer risk factors as well as its association with cannabis sales, policies, and use in the general population [18–20].

To our knowledge, no prior study has explored whether cannabis use differs by SDI among cancer patients. Our findings imply disparities in cannabis use by residential environment. For example, this study found that cancer patients residing in deprived areas use cannabis at higher rates than those in less deprived areas. This result is supported in the literature regarding other health behaviors pertinent to cancer patients, including increased tobacco and alcohol use [21, 22], lower HPV vaccine uptake [23], and lower cancer screening behaviors [24] in areas of greater residential deprivation. Additional research is needed in differences in cannabis use by SDI, including the need for additional symptom management/supportive care and opportunities for patient and clinician education on cannabis.

Results also found that individuals in more deprived areas reported significantly lower rates of medical marijuana prescriptions. We posit several potential explanations for this observation, including costs associated with obtaining and using a medical marijuana card, limited dispensary availability in more rural areas, and a multi-step application process. When the medical marijuana program started in 2018, patients in Ohio had delayed access to cannabis due to the limited number of certified physicians who could provide prescriptions [25]. Additional information is needed to better understand the decision-making by cancer patients residing in differing environments to attain a medical marijuana prescription compared to informal means as well as the facilitators and barriers to attaining cannabis products in cannabis dispensaries.

The current study also found that daily opioid use was nearly double among cancer patients in more deprived areas than those in less deprived areas. This finding is supported by a 2024 study using nationwide, county-level general population data that reported counties with more deprivation were significantly associated with a higher incidence of opioid dispensing rate [26]. In a study examining opioid use among cancer patients, those living in the least deprived SDI quintile received fewer opioids compared to those decedents living in most deprived SDI quintiles [27]. More research is warranted to determine influences behind higher rates of cannabis and opioid use among cancer patients living in deprived areas regarding resource allocation, symptom management, prescribing rates, and healthcare access and utilization.

This descriptive study has several important limitations. As with any cross-sectional design, selection bias (participation based upon cannabis use in particular) is a concern. The study is additionally limited by its ability to generalize findings across racial and ethnic minorities, as most participants were non-Hispanic and White. Lastly, we estimated SDI scores based upon residential mailing ZIP codes. SDI scores directly correspond to ZIP Code Tabulation Areas (ZCTA), created by the US Census Bureau. ZCTA approximate mailing ZIP codes but they do not always perfectly align, thus resulting in error. The study’s strengths include the novel use of two measures of residential environment, as indicated by urbanicity and areal deprivation, a large sample size, clinical chart review for eligibility confirmation, and detailed examination of self-reported cannabis and medication use. As with any descriptive study, our findings are hypothesis-generating and lack the capacity to explain why a disparity may exist. Rather, this study represents a preliminary step in identifying potential differences in cannabis and opioid prevalence by residential environment and suggests the need for more comprehensive investigations in larger, more representative populations.

## Conclusion

This descriptive study observed higher use of cannabis and opioids among cancer patients residing in areas of greater socioeconomic deprivation. Future exploration into these initial findings is warranted including motivation for cannabis use, history of cannabis use, concurrent use or replacement of opioids, patient education about cannabis, and palliative care access among diverse cancer patient populations.

## Supplementary Information

Below is the link to the electronic supplementary material.

Supplementary file1 (DOCX 20 KB)

## Data Availability

Datasets generated during the analysis of the current study are available from the corresponding author upon reasonable request.

## Abbreviations

RUCA: Rural–urban commuting area
SDI: Social deprivation index
ZCTA: ZIP code tabulation area
