# Supplementary material for: Prevalence of cannabis and medication use by indices of residential urbanicity and deprivation among Ohio cancer patients
Source: Cancer Causes Control. 2025 Feb 12;36(7):719–24. doi: 10.1007/s10552-025-01972-x (PMC12103373; doi:10.1007/s10552-025-01972-x)
Supplement: Supplementary file 1 — Supplementary file1 (DOCX 20 KB) [file 10552_2025_1972_MOESM1_ESM.docx]

**Supplemental Table 1.** Sociodemographic and clinical characteristics of study participants residing in Ohio, stratified on indices of urbanicity and socioeconomic deprivation.

|  |  | **RUCA, n (%)** | |  | **SDI, n (%)** | |
| --- | --- | --- | --- | --- | --- | --- |
| **Characteristic** | **Overall (n=854)** | **Metropolitan**  **(1-3; n=568)** | **Non-metropolitan**  **(≥4; n=286)** |  | **Less deprivation**  **(≤37; n=445)** | **More deprivation**  **(>37; n=401)** |
|  |  |  |  |  |  |  |
| Age, years; mean (SD) | 61.50 (11.93) | 62.00 (11.77) | 60.52 (12.21) |  | 61.61 (11.18) | 61.27 (12.71) |
|  |  |  |  |  |  |  |
| Sex |  |  |  |  |  |  |
| Female | 475 (55.95) | 326 (57.80) | 149 (52.28) |  | 250 (56.69) | 220 (55.00) |
| Male | 374 (44.05) | 238 (42.20) | 136 (47.72) |  | 191 (43.31) | 180 (45.00) |
|  |  |  |  |  |  |  |
| Race |  |  |  |  |  |  |
| Asian | 8 (0.95) | 8 (1.43) | 0 (0.00) |  | 5 (1.13) | 3 (0.76) |
| Black | 61 (7.22) | 59 (10.54) | 2 (0.70) |  | 12 (2.71) | 47 (11.90) |
| Other | 24 (2.60) | 15 (2.68) | 7 (2.46) |  | 11 (2.49) | 10 (2.53) |
| White | 754 (89.23) | 478 (85.36) | 276 (96.84) |  | 414 (93.67) | 335 (84.81) |
|  |  |  |  |  |  |  |
| Education |  |  |  |  |  |  |
| ≤ High school diploma | 255 (30.25) | 144 (25.76) | 111 (39.08) |  | 96 (21.87) | 156 (39.29) |
| Some college | 279 (33.10) | 178 (31.84) | 101 (35.56) |  | 131 (29.84) | 145 (36.52) |
| College or graduate degree | 309 (36.65) | 237 (42.40) | 72 (25.35) |  | 212 (48.29) | 96 (24.18) |
|  |  |  |  |  |  |  |
| Recruiting oncology clinic |  |  |  |  |  |  |
| Breast | 153 (17.92) | 105 (18.49) | 48 (16.78) |  | 80 (17.98) | 70 (17.46) |
| Dermatology | 86 (10.07) | 53 (9.33) | 33 (11.54) |  | 41 (9.21) | 45 (11.22) |
| Gastrointestinal | 145 (16.98) | 104 (18.31) | 41 (14.34) |  | 80 (17.98) | 64 (15.96) |
| Genitourinary | 88 (10.30) | 56 (9.86) | 32 (11.19) |  | 50 (11.24) | 38 (9.48) |
| Gynecology | 82 (9.60) | 48 (8.45) | 34 (11.89) |  | 50 (11.24) | 30 (7.48) |
| Hematology | 92 (10.77) | 64 (11.27) | 28 (9.79) |  | 47 (10.56) | 44 (10.97) |
| Otolaryngology | 28 (3.28) | 17 (2.99) | 11 (3.85) |  | 11 (2.47) | 17 (4.24) |
| Thoracic | 180 (21.08) | 121 (21.30) | 59 (20.63) |  | 86 (19.33) | 93 (23.19) |
|  |  |  |  |  |  |  |
| Treatment received |  |  |  |  |  |  |
| Currently receiving | 682 (79.95) | 452 (79.72) | 230 (80.42) |  | 359 (80.67) | 316 (79.00) |
| Completed | 136 (15.94) | 90 (15.87) | 46 (16.08) |  | 73 (16.40) | 62 (15.50) |
| Not yet received | 32 (3.75) | 23 (4.06) | 9 (3.15) |  | 12 (2.70) | 20 (5.00) |
| Do not plan to receive | 3 (0.35) | 2 (0.35) | 1 (0.35) |  | 1 (0.22) | 2 (0.50) |
|  |  |  |  |  |  |  |
| Spread of disease |  |  |  |  |  |  |
| Local | 422 (49.41) | 287 (50.53) | 135 (47.20) |  | 217 (48.76) | 200 (49.88) |
| Regional | 108 (12.65) | 65 (11.44) | 43 (15.03) |  | 59 (13.26) | 48 (11.97) |
| Distant | 138 (16.16) | 93 (16.37) | 45 (15.73) |  | 72 (16.18) | 65 (16.21) |
| Do not know | 186 (21.78) | 123 (21.65) | 63 (22.03) |  | 97 (21.80) | 88 (21.95) |
| Abbreviations: RUCA, Rural-Urban Commuting Area; SDI, Social Deprivation Index | | | | | | |
